# Advances in Basic and Translational Research as Part of the Center for the Study of Complex Malaria in India

**DOI:** 10.4269/ajtmh.21-1333

**Published:** 2022-10-13

**Authors:** Jane M. Carlton, Alex Eapen, Anne Kessler, Anupkumar R. Anvikar, Angelika Hoffmann, Om P. Singh, Steven A. Sullivan, Sandra Albert, Praveen K. Sahu, Sanjib Mohanty, Samuel C. Wassmer

**Affiliations:** ^1^ Center for Genomics and Systems Biology, Department of Biology, New York University, New York, New York;; ^2^ Department of Epidemiology, School of Global Public Health, New York University, New York, New York;; ^3^ National Institute of Malaria Research, Indian Council of Medical Research, IDVC Field Unit, National Institute of Epidemiology Campus, Chennai, Tamil Nadu, India;; ^4^ National Institute of Biologicals, Ministry of Health and Family Welfare, Government of India, Noida, Uttar Pradesh, India;; ^5^ University Institute of Diagnostic and Interventional Neuroradiology, University Hospital Bern, University of Bern, Switzerland;; ^6^ National Institute of Malaria Research, Indian Council of Medical Research, Dwarka, Delhi, India;; ^7^ Indian Institute of Public Health Shillong, Shillong, Meghalaya, India;; ^8^ Martin Luther Christian University, Shillong, Meghalaya, India;; ^9^ Department of Molecular and Infectious Diseases, Community Welfare Society Hospital, Rourkela, Odisha, India;; ^10^ Department of Infection Biology, London School of Hygiene and Tropical Medicine, London, United Kingdom

## Abstract

The Center for the Study of Complex Malaria in India (CSCMi) is one of 10 International Centers of Excellence in Malaria Research funded by the National Institutes of Health since 2010. The Center combines innovative research with capacity building and technology transfer to undertake studies with clinical and translational impact that will move malaria control in India toward the ultimate goal of malaria elimination/eradication. A key element of each research site in the four states of India (Tamil Nadu, Gujarat, Odisha, and Meghalaya) has been undertaking community- and clinic-based epidemiology projects to characterize the burden of malaria in the region. Demographic and clinical data and samples collected during these studies have been used in downstream projects on, for example, the widespread use of mosquito repellants, the population genomics of *Plasmodium vivax*, and the serological responses to *P. vivax* and *Plasmodium falciparum* antigens that reflect past or present exposure. A focus has been studying the pathogenesis of severe malaria caused by *P. falciparum* through magnetic resonance imaging of cerebral malaria patients. Here we provide a snapshot of some of the basic and applied research the CSCMi has undertaken over the past 12 years and indicate the further research and/or clinical and translational impact these studies have had.

## INTRODUCTION TO THE OVERALL THEME AND GOALS OF THE INDIA ICEMR

The Center for the Study of Complex Malaria in India (CSCMi), one of 10 International Centers of Excellence in Malaria Research (ICEMRs) located in malaria-endemic regions of the world, was launched in 2010.^1^ Unlike countries in the African subcontinent where *Plasmodium falciparum* is the dominant species and is transmitted by the major vector *Anopheles gambiae*, malaria in India can be caused by at least four parasite species (*Plasmodium falciparum*, *Plasmodium vivax*, *Plasmodium malariae,* and *Plasmodium ovale*), reflect coinfections of two or more species or genotypes, and can be transmitted by at least six *Anopheles* species, including *An. stephensi*, *An. fluviatilis*, *An. culicifacies*, *An. minimus*, *An. baimaii*, and *An. annularis*. The overall goal of CSCMi is to address major gaps in our understanding of such “complex malaria” and to elucidate how it affects the epidemiology, transmission, drug resistance, parasite population genomics, and pathogenesis of malaria in India. Key questions that we have attempted to answer include the following: What is the eco-epidemiology and transmission of complex malaria at our field sites and how is it changing? What is the role of environmental conditions in determining malaria transmission intensity in different eco-epidemiological contexts? And how genetically diverse is *P. vivax* and how does this compare with *P. falciparum*?

Twelve years later our research questions remain highly relevant. According to the WHO, India has had the largest absolute reductions in malaria cases in Southeast Asia, from 19.7 million in 2000 to 5.6 million in 2019, although it still contributed 86% of malaria cases in the region that year.^2^ The decrease in burden has changed the landscape of epidemiology and transmission in India, which in turn may influence the clinical presentation of malaria due to reduced exposure and a reduction in the resulting immunity.^3^ In the case of *P. falciparum*, a drop in incidence may be accompanied by a rise in the proportion of acute cases,^4^ and studies investigating the pathogenesis of severe malaria are thus crucial to inform new adjunctive therapies and improve clinical outcomes. In addition, in *P. falciparum*/*P. vivax* coendemic areas of the world, where the burden of the former has been reduced by control programs, a rise in the proportion of malaria caused by the latter has been reported.^5^ Vivax malaria has often been considered benign, but reports demonstrating severe complications arising from *P. vivax* infection challenge this assumption.^6^ Further studies are warranted to evaluate the extent of vivax epidemiology, and malaria-associated delayed morbidity and indirect mortality.^7^

The multidisciplinary partnership between Indian and international institutions under the umbrella of the CSCMi has advanced both basic and translational research, from updating epidemiology in malaria endemic states with distinct ecological and ethnicity profiles, to generating the first whole genome sequences of Indian malaria parasites and deciphering molecular mechanisms leading to the development of cerebral malaria. The dynamic and adaptive design of the CSCMi has enabled rapid responses to new challenges and allowed the development of spinoff projects such as a study of the etiology of nonmalaria febrile illness (e.g., Dengue, chikungunya, and scrub typhus) in a hospital setting in Rourkela, Odisha.^8^ Capacity building and technology transfer through access to international collaborations and training workshops are other essential components of the CSCMi, through which we have introduced state-of-the-art research platforms and facilities in endemic areas (for example, novel neuroimaging protocols, micro-electroencephalogram devices, next generation sequencing platforms, and high-throughput immunoassays).

**Figure 1.**
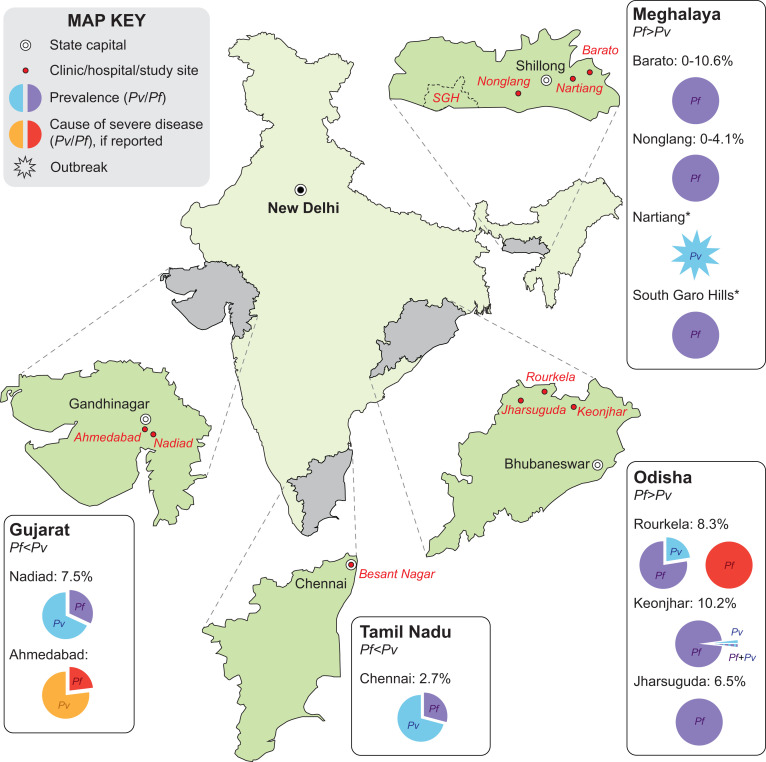
Malaria epidemiology at the Center for the Study of Complex Malaria in India sites in India, 2012–2021. Baseline epidemiology of *Plasmodium falciparum* (*Pf*) and *Plasmodium vivax* (*Pv*) was assessed in four states highlighted in grey through surveys undertaken at clinics/hospitals and in the community. Data are shown for: Gujarat (Ahmedabad^57^ and Nadiad^10^), Tamil Nadu (Chennai^10^), Meghalaya (Nonglang and Barato^12^^,^^13^), and Odisha (Rourkela and surrounding villages,^10^^,^^17^^,^^20^^,^^45^^–^^47^^,^^49^^,^^53^ and Jharsuguda and Keonjhar districts^9^). Pie charts in the inserts show *Plasmodium* species prevalence and species associated with severe disease for regions surveyed in each state; final data from two sites (1) Nartiang (*P. vivax* outbreaks monitored 2019–2021) and (2) South Garo Hills (a *P. falciparum* endemic district monitored 2020–2021, abbreviated SGH) are pending and indicated with an asterisk (*).

Here we outline some of the malaria research in India that has been undertaken by the Center over the past 12 years. The initial research theme of the Center from 2010 to 2017 centered on the study of complex malaria with respect to eco-epidemiological profiles, transmission and vector capabilities, and potential impact on drug resistance using genomic technologies, at three field sites in the east (Odisha with predominantly *P. falciparum* in forest/riparian ecology), in the south (the urban city of Chennai in Tamil Nadu, predominantly *P. vivax*), and in the west (the town and surrounding villages of Nadiad in Gujarat State with both *P. vivax* and *P. falciparum*) (Figure 1); see Das et al.^1^ for more details. A subsequent Special Project and Immunology Supplement award enabled the Center to expand its studies to the pathogenetic mechanisms involved in severe and cerebral falciparum malaria using magnetic resonance imaging technology at the Odisha field site. An Administrative Core, Genomics Core, and a Data Management, Biostatistics and Bioinformatics Core supported the research projects. From 2017 to date, studies have continued on severe and cerebral falciparum malaria at our Odisha site, while expanding to studies on the eco-epidemiology of vivax and falciparum malaria in new field sites of the northeastern state of Meghalaya. A second supplement award has enabled the Center to expand into 1) ongoing sociobehavioral and social network–based observational studies in the tribal villages of Meghalaya to determine what types of malaria-preventive interventions reduce malaria through good coverage and use and to identify demand and/or supply side barriers to malaria control measures; and 2) to undertake a randomized clinical trial evaluating the effectiveness of “malaria camps” implemented by the government of Odisha as part of India’s pivot to malaria elimination.^9^ Both these studies leverage the baseline data, infrastructure, and capacity established by the India ICEMR during the past 12 years. In what follows, we summarize the major scientific findings of several of these research projects and describe their clinical impact and translational value where apparent and/or further research is suggested (summarized in Table 1). Due to space limitations, we cannot describe each publication in detail, and the reader is referred to the original publication for further information.

**Table 1:** Summary of the clinical impact and translational aspects of some of the research projects undertaken by the Center

Research area, reference	Research finding	Clinical impact and/or translational aspect and/or further research suggested
**Epidemiology**		
Use of mosquito repellents^17^	Mosquito repellants (vaporizers, coils, creams) widely used in India, influenced by socioeconomic status, and not always associated with less malaria	Further clinical testing and evaluation of safety necessary for an evidence-based public health recommendation about how to choose and use repellents
Male behavior a risk factor for malaria^18^	Repellents used by ∼30% of 234 households in Sundargarh district, Odisha, but insecticide creams not used at all	Promotion of repellent cream use by at-risk groups could be explored in addition to mass screen or treat programs
Malaria symptoms and asymptomatic infections^16^	Many differences in complaints and symptoms between our sites, and factors associated with asymptomatic *Plasmodium* infections	The addition of the symptoms “headache,” “aches,” and “chills” to fever improved the malaria case-definition Malaria and asymptomatic infections differ by region, indicating that malaria elimination will require localized approaches
Reactive case detection^20^	RCD in areas of low malaria transmission and/or *Plasmodium vivax* is a labor-intensive strategy, and its benefit is not clear	Further studies are needed to assess how RCD can be optimized or to determine alternatives where interventions are targeted to family members, hotspots, or using serological markers (SeroTAT)
**Transmission**		
*Anopheles stephensi* breeding habitat^29^	Wells and overhead tanks are major breeding sources of the urban vector *An. stephensi* in Chennai	Overhead tanks as potential vector breeding sites could be targeted for intensified vector intervention measures
Zoophilic vectors^24^	Shift in vector species toward increased zoophilic behavior in recent years; modeling of regions dominated by zoophilic vectors indicate existing vector control tactics will be insufficient to achieve elimination	Cattle sheds could be targeted by control methods to focus on zoophilic behavior of *Anopheles* mosquitoes
**Genome-wide studies**		
*P. falciparum* and *P. vivax* whole genome sequences^38^	*P. vivax* exhibits twice as much genetic diversity than *P. falciparum*, suggesting a more stable and older association of this species with humans and suggests an increased capacity for functional variation in the global *P. vivax* population	*P. vivax* will be the more difficult species to eliminate.
*P. vivax* population genomics^39^	Analysis of 182 clinical isolate genomes from 11 countries identified signals of natural selection suggesting that *P. vivax* is evolving in response to antimalarial drugs, adapting to regional differences in the human host and mosquito vector	*P. vivax* has the more variable epidemiology, requiring greater sampling of *P. vivax* in different endemic regions to capture the standing genetic variation
Antibody responses to genome-wide *Plasmodium* antigens^22^	515 *P. vivax* and 500 *P. falciparum* antigens assayed with 353 plasma samples identified most immunogenic antigens of both species and *P. falciparum* antigens associated with asymptomatic infections	Range of immune responses characterized in different endemic settings in India argues for targeted surveillance approaches tailored to the diverse epidemiology
**Pathogenesis**		
Clinical characterization of CM^45^	Posterior reversible encephalopathy syndrome is associated with a reversible nature of the blood–brain barrier and is often associated with nonfatal CM	Evaluation of compounds aimed at reversing vasogenic edema is needed to provide potential new adjunct therapies
CM pathology^53^	Fatal CM is associated with severe brain swelling in children and with global brain hypoxia in adults	Adjunctive treatment highly likely to differ between the two age groups based on the predominance of cytotoxic edema in adults; focus should be made on decreasing brain hypoxia in adults
CM pathogenesis^60^	Endothelial protein C receptor-binding PfEMP-1 variants induce endothelial cell swelling and disrupt the blood-brain barrier in CM	Parasite ingress into brain endothelium is a contributing factor to the pathology of human CM
CM diagnosis/prognosis^46^	hsa-miR-3158-3p represents a promising biomarker candidate for CM prognosis across age groups	hsa-miR-3158-3p may be considered instead of neuroimaging to diagnose and monitor disease progression
CM pathogenesis^48^	CM-associated brain swelling has common determinants in both African and Indian populations	Adjunct therapies targeting brain swelling in CM patients may be effective in both children and adults, through restoration of normal function of the cytoprotective APC-EPCR signaling pathway
Clinical definition of CM^52^	Brain changes are frequent in *P. falciparum* infection, irrespective of the presence of coma	Spectrum of “cerebral” malaria is wider than initially thought; development of neurological sequelae in both uncomplicated malaria and severe noncerebral malaria groups must be evaluated

CM = cerebral malaria; RCD = reactive cased detection.

## DETERMINING BASELINE EPIDEMIOLOGY IN FOUR STATES OF INDIA

Our initial projects elucidated the baseline epidemiology of regions in four states of India (Gujarat, Meghalaya, Odisha, and Tamil Nadu) that differ by climate, ecology, and ethnic make-up (Figure 1), through census, cross-sectional, cohort, and clinic-based studies. We use polymerase chain reaction (PCR)-based methods as the gold standard for parasite detection, a strategy few other studies in India have used. At each visit, subjects who are enrolled after giving informed consent complete an extensive clinical questionnaire and supply a finger-prick blood sample for a rapid diagnostic test (RDT), microscopy, species-specific PCRs, and serology studies. If they are RDT positive, a vacutainer of venous blood is taken for downstream subprojects. Because these baseline epidemiology studies are observational only, patients diagnosed with malaria parasites by RDT or microscopy are treated by the malaria control authorities as per the National Vector Borne Disease Control Program malaria treatment guidelines. Here we describe some of our findings.

### *Plasmodium* infections in India are increasingly asymptomatic and submicroscopic.

A major revelation of our cross-sectional studies in Gujarat, Odisha, and Tamil Nadu during 2012–2015 has been the number of asymptomatic and submicroscopic infections. For example, 71% of infections in cross-sectional surveys in Chennai, Tamil Nadu, were asymptomatic, and 71% of infections were submicroscopic.^10^ Our more recent surveys in the northeastern state of Meghalaya in 2018–2019, where malaria prevalence has decreased dramatically over the past 5 years,^11^ present a similar picture, with 97% of all infections being asymptomatic and all submicroscopic,^12^^,^^13^ concordant with other studies in the northeast.^14^ As transmission declines across India, *Plasmodium* infections are increasingly associated with few symptoms and low parasite biomass, making diagnosis challenging and underscoring the need for more sensitive diagnostic tests at the point of care. Recently, it was proposed that facilities created for COVID-19 diagnosis—including PCR assays in field-friendly formats deployed across the country—can easily be coopted and harnessed for malaria diagnosis.^15^ Indeed, our own ongoing studies include implementation of molecular field assays in community health centers as part of a randomized clinical trial to evaluate Odisha State malaria camps. We also undertook an analysis of complaints and symptoms of 3,031 participants in our studies in Gujarat, Odisha, and Tamil Nadu, together with factors associated with asymptomatic *Plasmodium* infections. We found that addition of the symptoms “headache,” “aches,” and “chills” to “fever” improved the case definition of symptomatic malaria. Our findings indicate that malaria and asymptomatic infections differ by region in India, indicating that eliminating malaria will require localized approaches.^16^

### Mosquito repellents are commonly used in India but need more evaluation.

Our household census data in Gujarat, Odisha, and Tamil Nadu showed that the use of mosquito repellents such as coils, vaporizers, and mats was common and influenced by education level and socioeconomic status, but was not consistently associated with a reduction in malaria in our cross-sectional and clinic data.^17^ The market for repellents in India is considerable, but it is not clear if the different types of products are worth the investment, so more clinical testing and safety evaluation of these methods is warranted for an evidence-based recommendation of repellents and their role in elimination programs. In our follow-up study in Odisha that showed repellents used by ∼30% of households, insect repellant creams were not used at all,^18^ despite the finding that creams containing DEET have so far provided the longest protection in laboratory studies.^19^ The promotion of repellent cream use by at-risk groups could be further explored in addition to mass screen-and-treat programs in high-risk villages.

### Testing novel surveillance tools as malaria transmission in India wanes.

We have piloted novel strategies for malaria surveillance and elimination at several of our sites. In 2014, our reactive case detection (RCD) studies at two urban areas with low prevalence of mainly *P. vivax* used PCR to detect *Plasmodium* parasites in 131 contacts of 20 index cases (Nadiad, Gujarat) and 868 contacts of 18 index cases (Chennai, Tamil Nadu), a strategy that identified only four new infections at the latter site. RCD thus proved to be a labor-intensive strategy that was not useful at our vivax endemic sites.^20^ Further studies are needed to assess whether RCD can be used in such settings—for example, by using serological markers for detecting recent *P. vivax* infection that could indicate hypnozoite carriers to be targeted for treatment with antihypnozoite drugs (a strategy called SeroTAT).^21^ We have also undertaken pilot studies of serological responses to *P. vivax* and *P. falciparum* at all four of our sites in India, using either ∼1,000 *Plasmodium* antigens spotted on microarray chips^22^ (the same microarrays as used by several other ICEMRs, allowing for comparison of these pilot studies between sites), or a high-throughput bead-based assay with 17 *Plasmodium* antigens that reflect past or present exposure.^13^ Our studies in Gujarat, Odisha, and Tamil Nadu identified the most immunogenic *Plasmodium* antigens and *P. falciparum* antigens associated with asymptomatic infections.^22^ In Meghalaya, analysis of serological exposure markers at two sites found that responses increased with age, providing further evidence of a decrease in transmission in this area.^13^ We continue to use the bead-based assay with a refined panel of *Plasmodium* antigens to classify exposure as either recent or long term in studies investigating a recent *P. vivax* outbreak in Jaintia Hills, Meghalaya, and as part of the malaria camps effectiveness trial mentioned earlier.^9^ Seroepidemiology is a promising approach both for determining the changing epidemiology of a region and for targeted malaria control and elimination interventions.^23^

## TRANSMISSION AND VECTOR STUDIES

Our transmission and vector studies in Tamil Nadu, Odisha, and Meghalaya have included surveillance projects of *Anopheles* species in the same villages as our epidemiology studies, quantifying the role of environmental conditions in determining malaria transmission intensity, and identifying novel mutations associated with insecticide resistance that key Indian *Anopheles* vectors have developed.

### *Anopheles* surveillance.

Given the diversity of *Anopheles* mosquitoes in India, an important component of the CSCMi has been capturing *Anopheles* adults using CDC light traps and aspirators, accompanied by morphological and molecular classification, salivary gland and midgut dissections for malaria parasite detection, and blood meal analysis. One surveillance study in 15 villages in Odisha during 2012–2013 confirmed *An. culicifacies* and *An. fluviatilis* as the major vectors in this area, with greater densities found in cattle sheds compared with human dwellings.^24^ We found a shift from the strongly anthropophilic *An. fluviatilis* S-type to the more zoophilic T-type with a preference of cattle sheds over human dwellings (as described in other studies)^25^; such a shift in zoophilic behavior could be a response to the intensified use of indoor residual spraying (IRS) and long-lasting insecticide-treated nets (LLINs) in the area. Blood meal analysis indicated possible frequent switching of feeding between humans and animals, and our modeling studies suggested that such a zoophilic cycle was unlikely to be affected by scaling up conventional tools such as IRS or LLINs. Alternatively, redirecting control efforts toward the zoophilic cycle could put the elimination threshold within reach. A year-long survey of cattle sheds and human dwellings in Chennai identified *An. stephensi* as greatly preferring cattle sheds to human dwellings for both resting and feeding, and those found in human dwellings greatly preferring thatched structures.^26^ We also reported the first detection of malaria parasites in *Anopheles subpictus*, not previously considered a human malaria vector in this city.^26^ In Meghalaya, our surveillance studies during 2018–2019 identified ∼13 *Anopheles* species, some of which are known malaria vectors, including *Anopheles jeyporiensis*.^12^ Absence of *An. baimaii* and *An. minimus* corroborated recent reports from northeast India that these forest-associated mosquito species are in decline.^27^ Further studies are needed to determine which species are now contributing to malaria transmission and to characterize their biological attributes relevant to vector control, including biting-time, host-preference, larval ecology, and seasonal abundance.

### Environmental conditions determining malaria transmission.

We monitored temperatures in a range of indoor and outdoor resting habitats of mosquitos in two urban slum sites at our Chennai field site because standard estimates of environmental temperature derived from the local weather stations do not provide realistic measures of temperatures within actual transmission environments, and even small differences in mean temperatures or diurnal temperature ranges could lead to large variations in mosquito and/or parasite life history traits that determine transmission intensity.^28^ A year-long weekly study of the available clear/clean water mosquito breeding habitats, such as wells, cement cisterns, plastic barrels, and overhead tanks (OHT) found that OHTs were the predominant breeding habitat for the local malaria vector *An. stephensi*, leading us to recommend directing intervention efforts to that habitat.^29^ The presence of fluoride correlated with *An. stephensi* immature density, an indicator/predictor of vector breeding.^30^ Cattle sheds are the preferred resting place of *An. stephensi*, and dawn is the optimal time to collect and estimate its densities.^31^

### Molecular mechanisms of insecticide resistance in Indian *Anopheles*.

Our initial studies involved detection of “knockdown resistance” (*kdr*) in Indian mosquito species due to mutations in the voltage-gated sodium channel gene that confers resistance against DDT and the pyrethroid group of insecticides. We reported two alternate point mutations leading to the same amino acid substitution (L1014F) in *An. subpictus* and developed a new assay that detected the mutations at high frequency (82%) in the Indian study area.^32^ We also mapped the distribution of two *kdr* mutations, L1014F and L101S, in *An. stephensi*^33^ as well as *An. culicifacies*^33^ in different Indian populations. In another study, we studied the evolution of the DDT-resistance mechanism in *An. stephensi* under laboratory conditions and identified tandem duplication of a genomic segment of the GSTe (epsilon class of glutathione S-transferases) gene array encoding GSTe2 and GSTe4, leading to increased transcription of these two genes. The duplication event also led to the diversification of these two genes, resulting in two paralogues of GSTe2 and three paralogues of GSTe4 being present in a single mosquito.^34^

## POPULATION GENOMICS AND ANTIMALARIAL DRUG RESISTANCE STUDIES

We have exploited advances in next generation sequencing (NGS) technology^35^ to study antimalarial drug resistance and population genomics of *P. falciparum* and *P. vivax* in India.

### An NGS facility in India and studies on antimalarial drug resistance.

We set up an NGS facility at the ICMR/National Institute for Malaria Research in Delhi^36^ and used it to develop an amplicon sequencing method for high-throughput deep sequencing of six full-length *P. falciparum* drug resistance genes (*Pfmdr1*, *Pfcrt*,* PfDHFR*, *PfDHPS*, *PfK13*, *Pfmrp1*).^37^ We identified known and novel mutations of these genes in patient samples collected from our epidemiology studies in Tamil Nadu, Gujarat and Odisha, for example, in *PfK13* associated with artemisinin resistance, as well as heterozygous loci containing allelic variants, indicative of mixtures of resistant and sensitive clones in the same individual. Any marker gene can be swapped into the panel, making this “amplicon-seq” method potentially suitable for studies of, for example, *P. vivax* drug resistance or the number of *Plasmodium* genotypes in an infection (“complexity of infection”).

### The first *P. falciparum* and *P. vivax* reference genomes from India.

One of our major achievements has been to generate the first *P. vivax* and *P. falciparum* reference genomes from India.^38^ Our population genomic analysis of six *P. vivax* strains from India, Latin America, North Korea, and Mauritania with six comparator *P. falciparum* lines revealed twice as much genetic diversity in *P. vivax*, suggesting a more stable and older association of this species with humans than for *P. falciparum*. In addition, the greater genetic diversity and gene family variability that we found suggests an increased capacity for functional variation in the global *P. vivax* population. We concluded that *P. vivax* malaria will likely be the more difficult parasite to eliminate.^38^

### *P. vivax* population genomics across ICEMRs.

We generated whole genome sequences of ∼180 global *P. vivax* clinical isolates from five of the ICEMRs,^39^ identifying diversity hotspots in the genome and the first *P. vivax* selective sweeps of two drug resistance-associated genes *PvDHR* and *PvDHPS*. Other signals of natural selection that we identified suggest that *P. vivax* is adapting to regional differences in the human host and mosquito vector. The several Indian *P. vivax* genomes from our Chennai site showed significant admixture with isolates from Africa and support the hypothesis that contemporary African and South Asian *P. vivax* populations are genetically similar, suggesting that the latter may have genetically mingled with European (“New World”) lineages during the colonial era.

## PATHOGENESIS OF SEVERE MALARIA

India is endemic for *P. vivax* and *P. falciparum*, which has allowed us to investigate severe malaria caused by both species in different eco-epidemiological settings.

### Severe falciparum malaria.

Severe falciparum malaria is a complex disease with a wide spectrum of manifestations^40^ and poorly understood pathogenesis. It affects sub-Saharan children and Southeast Asian adults but with different clinical presentations^41^: whereas African children predominantly develop life-threatening anemia, metabolic acidosis, and cerebral malaria (CM), severe malaria in Southeast Asian adults is characterized by a multiorgan system involvement,^3^^,^^42^ including brain (CM), kidneys (acute kidney injury), liver (jaundice), and lungs (acute respiratory distress syndrome).

#### Cerebral malaria.

**Figure 2.**
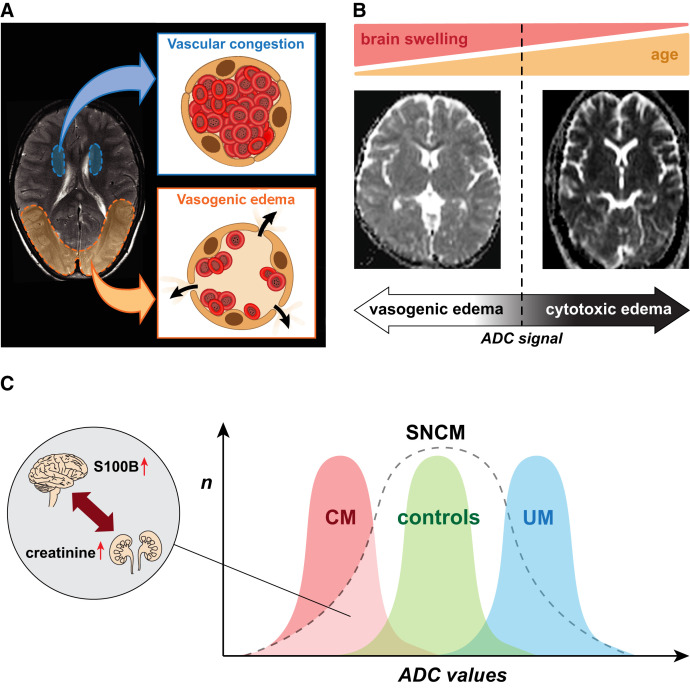
Summary of magnetic resonance imaging findings in India. (**A**) Schematic representation of the two main mechanisms leading to brain swelling in nonfatal Indian cerebral malaria (CM) patients—namely, reversible vasogenic edema with posterior predominance (redolent of posterior reversible encephalopathy syndrome), and vascular congestion in the basal ganglia (likely caused by the sequestration of *P. falciparum*–infected erythrocytes). (**B**) The cause of death in fatal CM differs with age: in children, severe brain swelling is associated with globally increased apparent diffusion coefficient (ADC) signal, indicative of diffuse vasogenic edema (left). In contrast, fatal CM in adults is associated with a profound, global hypoxia evidenced by the decreased ADC signal and mild or no brain swelling (right). (**C**) ADC value ranges in uncomplicated malaria (UM), healthy control, and CM cases overlap, and severe noncerebral malaria (SNCM) patients display a wide spectrum of ADC values. Extremes values in SNCM cases are similar to those seen in UM and CM; in the latter case, brain involvement is supported by elevated plasma S100B, a biomarker of brain injury, despite no WHO-defined coma. Low ADC values are strongly correlated with elevated plasma levels of creatinine in our cohort, indicative of acute kidney injury. Collectively, our findings suggest a brain–kidney pathogenic crosstalk in SNCM.

Approaches such as in vitro and in vivo models and autopsy studies have been used to unravel the mechanisms leading to the development of CM, but have provided limited answers and are inherently flawed.^3^ The recent use of neuroimaging has circumvented these obstacles and enabled imaging of pediatric and adult patients at both the acute stage of CM and also at follow-up for survivors.^43^^,^^44^ Our magnetic resonance imaging (MRI) project in the *P. falciparum*-endemic setting of Odisha, where both pediatric and adult CM cases occur, leveraged the availability of a 1.5-Tesla MRI scanner at Ispat General Hospital, Rourkela, allowing imaging comparisons between different age groups within the same cohort. We first analyzed brain changes in CM survivors and observed two distinct patterns of cerebral swelling in both adult and pediatric patients, often developing simultaneously in distinct parts of the brain. The occurrence of posterior vasogenic edema caused by an impairment of the blood–brain barrier that reversed upon treatment was indicative of posterior reversible encephalopathy syndrome, which we demonstrated for the first time in the context of CM (Figure 2A).^45^ Several patients in our cohort had concurrent cytotoxic edema in their basal nuclei with perfusion parameters indicative of vascular engorgement, likely to be caused by the sequestration of *P. falciparum*–infected erythrocytes (Figure 2A). Our results support the hypothesis that both endothelial dysfunction and microvascular obstruction make independent contributions to the pathogenesis of CM, providing opportunities for novel therapeutic interventions.^45^

In a second study, we investigated cerebral changes associated with fatality in both adults and children of the same cohort. We confirmed previous findings showing that severe brain swelling was the main feature associated with death in pediatric CM.^44^ Our results demonstrated for the first time that this swelling decreased with age and that fatal adult CM is associated with severe hypoxic injury (Figure 2B), suggesting that different adjunct therapies need to be considered according to the patient age.^53^ However, detecting these brain changes upon admission is not feasible in most clinics in endemic settings. Our team therefore assessed potential biomarker candidates associated with specific neuroimaging features in CM. We found that plasma levels of hsa-miR-3158-3p, a microRNA involved in hypoxia-related processes, are significantly higher in fatal CM compared with nonfatal cases and are associated with MRI features of fatal CM for both children and adults.^46^

Lastly, we leveraged the presence of an MRI scanner at a different ICEMR site in Blantyre, Malawi,^47^ to perform the first comparative blood and brain MRI profiling of its kind between our Indian cohort of pediatric and adult CM and a Malawian cohort of pediatric CM. We identified common determinants of brain swelling across the two study sites—namely, high parasite biomass determined by plasma PfHRP2 levels and elevated endothelial protein C receptor (EPCR)-binding *var* transcripts.^48^ These findings confirm the role of parasite biomass as a pivotal pathogenic mechanism in CM and further suggest that restoring the cytoprotective activated EPCR signaling pathway abrogated by sequestered parasitized erythrocytes may represent a promising therapeutic avenue for patients with CM.^49^^,^^50^

#### Severe noncerebral malaria.

The focus of the CSCMi MRI project was recently broadened to characterize brain changes in severe, noncerebral malaria patients (SNCM), a group first recruited to serve as control. Indeed, two earlier studies reported that mild to moderate brain swelling^43^ and/or parenchymal changes^51^ on MRI were common in severe malaria patients with or without CM. Using the same imaging protocol from our previous studies, we found that both uncomplicated malaria and SNCM patients show a wide pattern of cerebral changes compared with healthy controls, suggesting that the brain is frequently affected in *P. falciparum* infection.^52^ We showed that some SNCM patients had patterns of brain changes similar to CM despite the absence of coma, and these were strongly associated with the presence of acute kidney injury. Our findings not only indicate a kidney–brain pathogenic crosstalk but also that severe malaria leads to a spectrum of neurological findings, where CM is only defined by the presence of coma. This highlights the need to revise the existing definition of “cerebral” malaria because severity syndromes are overlapping (Figure 2C) and the current Glasgow Coma Score cutoff is not optimal to identify all cases with acute brain alterations. Indeed, although not technically defined as having cerebral involvement, SNCM patients can develop brain change patterns like those observed in CM. The short- and long-term impact on neurological and psychological functions have never been assessed in this patient group, and these are now warranted so that strategies to identify patients at risk and help their recovery and rehabilitation can be developed.

The CSCMi MRI project benefited greatly from the long duration of the funding, which allowed nurturing of pivotal international collaborations, ultimately facilitating reproducibility and validation of imaging results. It has also led to the development of a range of research and clinical tools available to the malaria community, including the design of hypotheses-driven imaging protocols and quantitative methods to assess brain changes in falciparum malaria infection, and to the establishment of plasma biomarkers that strongly correlate with MRI findings and can therefore be used for diagnostic and prognostic approaches. These include lipocalin-2,^53^ hsa-miR-3158-3p,^46^ and S100 calcium-binding protein B (S100B)^52^; an assessment of their use for case management at the point of care is currently under way. Lastly, the project generated clinical datasets and reports that can be used to inform novel adjunctive therapies in the future.

### Severe vivax malaria.

Evidence of *P. vivax*–associated morbidity and mortality has accumulated over the past decade,^54^ including delayed morbidity and indirect mortality,^55^ challenging the historic dogma of a benign illness. It is therefore important to evaluate rigorously the spectrum of disease caused by *P. vivax* in India, both at the acute and follow-up stages. Our first study in this area enrolled PCR-confirmed severe malaria patients at the Civil Hospital in Ahmedabad, Gujarat, followed over 12 months to compare morbidity and mortality between severe vivax and severe falciparum malaria.^56^ In this setting, severe vivax was found to be more frequent than severe falciparum malaria, jaundice was the most common complication of severe vivax malaria, and adults were predominantly affected rather than children, potentially indicating an age shift in antimalarial immunity. Although this may be a result of the recent decrease in transmission across India, further studies using serological evaluation of exposure markers to monitor the impact of elimination programs are warranted, as well as follow-up studies combining clinical characterization of severe vivax malaria with relapse and indirect mortality assessments.^57^^,^^58^

## SUMMARY AND CONCLUSIONS

Our research and discoveries during the 12 years of the CSCMi highlight what can be achieved with continuous, long-term, stable funding and underscore the importance of undertaking basic and translational research on this important disease. A key factor has been the adaptability of the Center in the face of the changing epidemiology of malaria in India, making it responsive to new developments and questions as they emerge. For example, our studies on severe vivax malaria in Ahmedabad, Gujarat, were precipitated by a notable increase in number of cases in this area in 2016–2017, reported to us by clinicians at BJMC Civil Hospital in Gujarat. A central component of the CSCMi is interaction with malaria research, policy, and control counterparts in India to ensure that the research is relevant, significant, and strategic and that it provides opportunities for training, capacity building, and transfer of new technology. Our efforts and interactions in this area are described in the accompanying paper.^59^
